# Universal health coverage, health systems strengthening, and the World Bank

**DOI:** 10.1136/bmj.j3347

**Published:** 2017-08-31

**Authors:** Marlee Tichenor, Devi Sridhar

**Affiliations:** University of Edinburgh, Edinburgh, UK

## Abstract

In the second article of the series, **Marlee Tichenor** and **Devi Sridhar** discuss how the World Bank is promoting better access to healthcare

**Key messages**

In the era of sustainable development goals, the World Bank plays a pivotal role in promoting universal health coverage and strengthening health systems. In 2010, the bank provided 32% of the global health systems support budget
The World Bank’s health policy focus has shifted from population control (1970s), to primary healthcare direct lending (1980-6), to health reform (1987-96), to the enhancement of healthcare systems (1997-2007), to a health systems approach (2007-present)
The World Bank has a comparative advantage over WHO to lead the universal health coverage agenda given its access to ministries of finance, its staff expertise in measurement, its broad multisectorial portfolio, and its lending power
The World Bank’s expanded role in global health carries with it the risks of further privatisation of the health sector and major tension between its mandate and the right to health at the heart of universal health coverage

The sustainable development goals (SDGs) formally promote universal health coverage as a key objective for the global health community. This focus on universal health coverage arose both from a desire to maintain the health gains achieved by the millennium development goals and from criticism that those goals were short term in nature and narrowly focused on disease specific approaches.^1^ ^2^

On 12 December 2012, universal health coverage had unequivocal endorsement from the UN General Assembly (including the United States) with the approval of a resolution that confirmed the “intrinsic role of health in achieving international sustainable development goals.”^3^ Also in 2012, a World Health Organization discussion paper on the post-2015 health agenda (what would emerge as the SDGs) identified universal health coverage as a “way of bringing all programmatic interests under an inclusive umbrella.”^4^ The 2014 Ebola virus outbreak struck in the middle of deliberations on the SDGs, and reviews of the global health community’s response to the epidemic have emphasised the importance of health systems strengthening to achieving equitable, accessible, and resilient healthcare.^5^ ^6^

In this paper, we examine the World Bank’s past and present role in health systems strengthening within the current global health architecture, and critically analyse several points this raises.

## The governance of health systems strengthening

Despite universal health coverage’s growing prominence, the term and its relationship to health systems strengthening are under debate.^7^ To better understand trends in the World Bank’s investment in health systems and its role in promoting universal health coverage, we must find workable definitions for the terms. The World Bank defines health systems as “the combination of resources, organisation, financing, and management that culminate in the delivery of health services to the population.” While WHO’s definition is “all activities whose primary purpose is to promote, restore, and maintain health.”^8^ ^9^ The term “health systems” has, in the last decade and a half, brought with it systems thinking. As a result, health systems—which are understood to include public and private sectors, communities and families, and health financing bodies and pharmaceutical companies, among other health related organisations—are understood to be “complex adaptive systems” that are continually reorganising in “both formal and informal ways” and unpredictably reacting to inputs into the system.^9^ ^10^

Within the SDGs, universal health coverage includes “financial risk protection, access to quality essential healthcare services, and access to safe, effective, quality, and affordable essential medicines and vaccines for all.”^11^ Health systems strengthening, then, is often taken to indicate the actions taken to achieve the goal of universal health coverage and is often framed as the horizontal approach in contrast to disease specific or vertical approaches.^12^ ^13^

Because the definition of a health system is different in different organisations, the means of strengthening it are similarly varied. The bank emphasises that strengthening health systems requires attending to the many actors, commodity chains, policies, and financing mechanisms that make up a country’s health system. To the bank, strengthening health systems involves “enhancing public-private partnerships,” “setting up the right payment mechanisms,” and “ensuring the right logistics,” among many other interventions.^9^

However, the bank’s approach is just one of several institutions in the global architecture (table 1, figures 1, 2, 3[Fig f1 f2 f3]). For example, in recent years the Global Fund to Fight HIV/AIDS, Tuberculosis, and Malaria, along with Gavi, have increasingly channelled funding to strengthening health systems, albeit with a restricted agenda tied to these three diseases, thereby promoting a diagonal approach that mirrors the 1980s movement of “selective primary healthcare” (table 1). WHO first focused on the right to health at its 1978 Alma-Ata Conference; in recent years, however, it has focused on technical support through creating better metrics for measuring progress towards universal health coverage and quality of strengthening health systems.^14^ The Bill and Melinda Gates Foundation is largely involved with the universal health coverage agenda through its work with WHO and the World Bank on their existing initiatives and supporting the Global Financing Facility at the World Bank.^15^

**Table 1 tbl1:** Major global organisations involved with the universal health coverage agenda^33^ ^34^ ^35^ ^36^ ^37^ ^38^

Name of organisation	HSS investment in 2013	Key historic milestones/partnerships	Current strategy	In-country contact	Relative strengths
WHO	$608m	1975: Primary healthcare strategy launched	UHC monitoring framework with the World Bank, based on two core components of UHC: coverage of the population with quality, essential health services; and coverage of the population with financial protection	In-country offices	Technical support
		1978: co-sponsors Alma-Ata International Conference with Unicef			
		1987: co-sponsors the Bamako initiative with Unicef	Primary healthcare performance initiative with World Bank and Gates Foundation	Ministries of health	UHC main priority of incoming director general
		2000: WHO health report on health systems			
		2005: World Health Assembly Resolution 58.33 on universal health coverage	UHC2030; UHC partnership; social health protection network		
World Bank	$1086m (This figure excludes the bank’s trust funds in HSS, which we estimate to be another $189m)	1980-6: primary healthcare direct lending	UHC monitoring framework with WHO (more above)	In-country offices	Technical support
		1986: *Financing Health Services in Developing Countries* document introduces user charges	Primary healthcare performance initiative with WHO and Gates Foundation	Ministries of finance	Access to ministers of finance
		1993: *World Development Report* emphasises essential services	Committing $15 billion 2016-20 to UHC in Africa		Capacity for and experience in sector-wide approaches
		2007: Healthy development emphasises importance of strengthening health systems; Health results innovation trust fund established	Global financing facility		Ability to leverage alternate means of finance
			UHC2030		
Unicef	$42m	Mid-1960s: requests that WHO invest in strengthening health systems	HSS are “actions that establish sustained improvements in the provision, utilisation, quality, and efficiency of health services, broadly defined to include family care, preventive services, and curative care, and that produce equitable health, nutrition, and development outcomes for children, adolescents, and women”	In-country offices	Local engagement
		1978: co-sponsors Alma-Ata Conference with WHO			
		1982: Unicef’s version of “selective primary healthcare,” growth monitoring, oral rehydration, breastfeeding, and immunisations (GOBI) launched			
		1987: co-sponsors Bamako initiative with WHO	Its “seven step approach to situation analysis and identification of priority actions in HSS” include identifying underserved groups and solutions to bottlenecks in coverage determinants	Various government departments such as National Agency for Statistics and Demography	Training of health workers
			UHC2030		
Gavi, the vaccine alliance	$144m	2005: Gavi board first takes steps to widen support of HSS, recognising that immunisation coverage is dependent on “strong service systems”	The second of Gavi’s four goals in its PHASE IV 2016-20 work is “the systems goal” to “increase effectiveness and efficiency of immunisation delivery as an integrated part of strengthened health systems”	No in-country presence	Uses vaccine distribution chains to strengthen health systems
		2009: commissioned review of Gavi’s health system strengthening support	UHC2030	Partners with UNICEF and WHO for in-country distribution	
Global Fund to Fight AIDS, Tuberculosis, and Malaria	$1246m	2007: Round 7 grants granted HSS support with disease specific component	The Global Fund’s $9bn commitment for 2017 through 2019 includes $3bn of investments in systems for health such as strengthened procurement systems and supply chains, improved data quality and data management systems, and strengthened human resources for health	No in-country presence	Uses investments in HIV/AIDS, TB, and malaria to strengthen health systems, mirroring the selective primary healthcare movement of the 1980s
			UHC2030	Country coordinating mechanism	
Bill and Melinda Gates Foundation	$100m ($146m through the other organisations listed above)	2013: publish a document that states that UHC “has much to commend it in terms of an aspirational goal” but “due to the lack of robust evidence of links between UHC . . . and the desired impact of improved health outcomes,” the Gates Foundation does not agree it should be included in the SDGs	Primary healthcare performance initiative with World Bank and WHO	No in-country presence	Extreme flexibility
			Major involvement with global financing facility; UHC2030	Variable, depends on country and project	

UHC=universal health coverage; HSS=health system strengthening.

**Figure f1:**
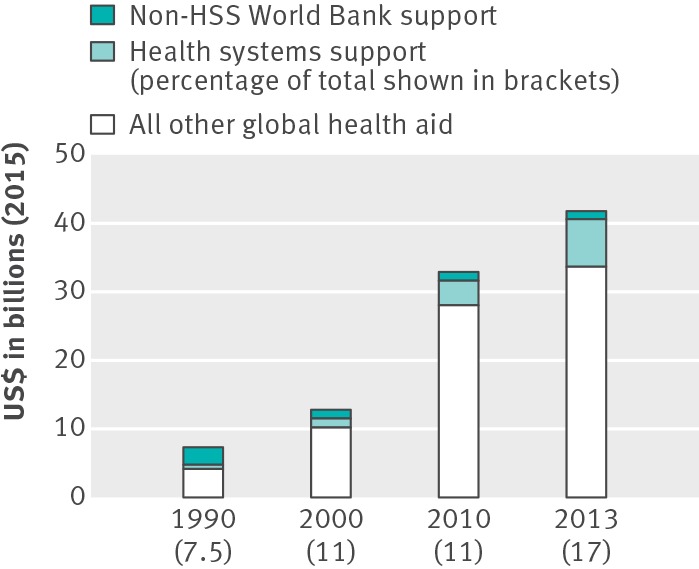
**Fig 1** Health systems support in development assistance for health^16^ ^17^ ^18^ ^19^(2013 is included because it is the most recent year with complete breakdowns for financial contributions on the Institute for Health Metrics and Evaluation website)

**Figure f2:**
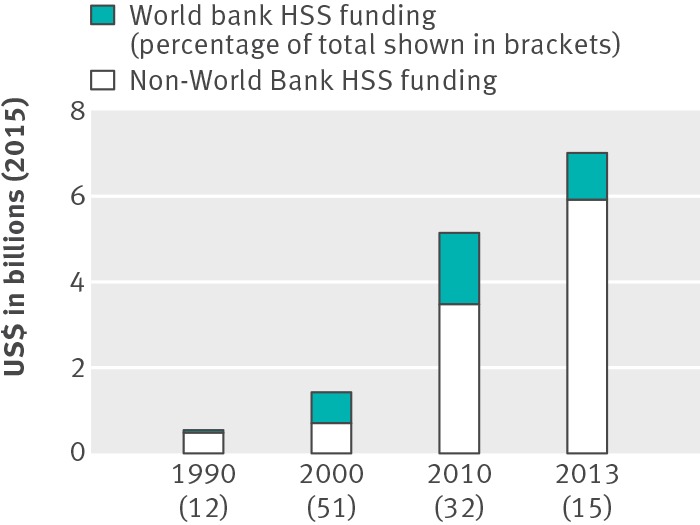
**Fig 2** Financial contribution of the World Bank to health systems strengthening^16^ ^17^ ^18^ ^19^ (2013 is included because it is the most recent year with complete breakdowns for financial contributions on the Institute for Health Metrics and Evaluation website)

**Figure f3:**
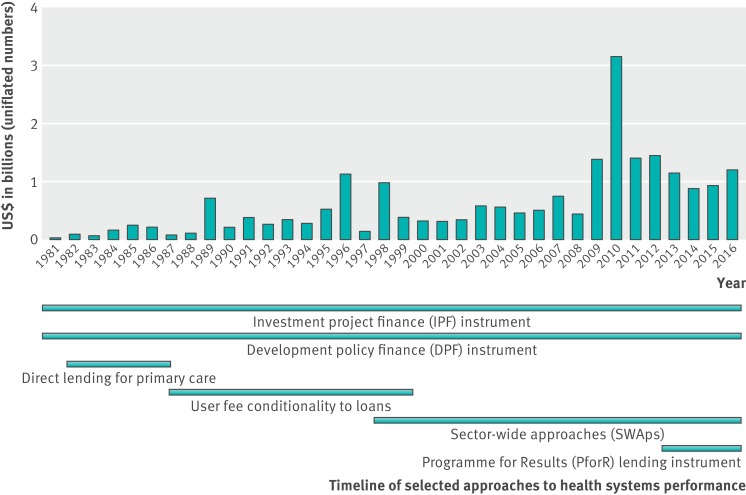
**Fig 3** World Bank commitments to health systems performance

## The World Bank’s history in strengthening health systems

Since its first funded health project in Jamaica in 1970, the World Bank has devoted much of its health financing to strengthening health systems, defining that elusive term here as a broad based, multisectorial, and infrastructural approach to developing health systems. Using the Independent Evaluation Group’s timeline, the bank’s policy focus has shifted from population control in the 1970s, to primary healthcare direct lending from 1980 to 1986, to health reform from 1987 to 1996, to the enhancement of healthcare systems from 1997 to 2007, to a health systems approach from 2007 to the present.^2^ ^13^

The inaugural health sector project in Jamaica marked the first trend in the bank’s policy focus in health: population control. Of the $2m (£1.6m; €1.8m) invested in the programme, 84% was spent on the expansion and remodelling of Jamaica’s major maternity hospital and the construction of 10 rural maternity centres. With important exceptions—like the Onchocerciasis Control Programme established in 1974^18^—the bank’s early interest in the health sector was rooted in its desire to “slow down population growth,” ease “population pressure,” and thereby mitigate its “heavy strain on government expenditures on education, health, and housing” in the global south.^20^ It was within this context of population control that the bank supported infrastructural and technical support of health services.

In 1981, the bank initiated the health and population project in Tunisia, which was arguably its first project explicitly promoting basic healthcare. The project corresponded with a policy shift away from population control towards tackling growing urban-rural disparities and emphasising a preventative approach to healthcare over a curative one. This shift towards primary healthcare was a part of international health development’s emphasis on “health for all” following the Alma-Ata Declaration of 1978. However, because of what the bank saw as systematic constraints that served as obstacles to providing more efficient and equitable health services, it restructured its approach to health sector funding, which is outlined in their 1986 policy document.^13^

It is in this document—which calls for health reform and more attention to the world’s poor—that the bank’s population, health, and nutrition department (HNP) introduced user charges as a means to equalise access to government run health services in developing countries. They argued that user charges could help make health systems more equitable, considering that the rich—who benefit most from public services—would have to pay. This would theoretically free up government resources that could be directed at programmes and facilities for the poor.^21^ Like WHO and Unicef’s Bamako Initiative after it, the introduction of user charges led to a further widening of health inequities in many contexts in the global south, producing results at odds with the policies’ intentions.^22^ ^23^

As a result of its health investment restructuring, the bank came to fund “umbrella” projects with other international health financiers at the end of the 1980s and in the early 1990s. These incorporated “capacity building” within larger projects on maternal health and nutrition. The Independent Evaluation Group has pointed out that this restructuring actually isolated bank funded projects from the health ministries of recipient countries and contributed to the fragmentation that had become increasingly part of global health aid.^24^ The sector-wide approach was introduced in 1997 as a means to “overcome inefficiencies, lack of government ownership, and a number of other problems” constraining international aid.^25^

The HNP’s 2007 *Healthy Development* policy document explicitly took on the work and language of health systems strengthening, and re-emphasised the connection between the bank’s goal of eradicating poverty with the financial constraints and risks that illness put on the world’s poor.^9^ Since 2007, the bank’s investment in strengthening health systems has included many new approaches to financing—for example, the Health Results Innovation Trust Fund, established in 2007; the lending instrument Programme for Results Financing established in 2011; and the recently launched Global Financing Facility. These are all mechanisms with which the bank aims to capture more resources for countries’ health systems for the promotion of equitable, financially accessible healthcare. With these mechanisms, the bank emphasises the importance of including private health services within a holistic view of strengthening health systems. The Programme for Results Financing was established as the third arm next to the bank’s longstanding investment project finance and development policy finance instruments (figure 3). Within the context of innovative financing, there have been some questions about the limits of results based financing, as the Health Results Innovation Trust Fund and Programme for Results Financing lent on the condition of countries’ ability to prove they met certain health indicators. The Global Financing Facility has explicitly not incorporated requirements for results based financing in its framework because of backlash.^15^

The bank’s current portfolio in strengthening health systems includes project loans, trust funds, partnerships, new lending instruments, and general budget support, among other modalities. Included within this portfolio are partnerships such as the Primary Health Care Performance Initiative and the International Health Partnership, which provide platforms for coordinating between multiple modes of defining, supporting, and measuring strengthening health systems. Along with the World Bank contributions discussed above, figure 3 provides a timeline of trends of when some approaches to strengthening health systems and lending instruments have been introduced and phased out.

## Benefits and risks of the bank’s expanding role

With its long standing interest and involvement in broad based health systems support and experience with innovative financing, the bank has strengths that can support universal health coverage. Firstly, because it has access to ministries of finance, the bank can push for universal health coverage in the way no other global health agency can. Secondly, because it is simultaneously a knowledge bank with expert staff, it can drive the development of metrics for monitoring progress towards universal health coverage. In 2015, as a means to monitor countries’ progress towards the goal, WHO and the bank published the first report on measuring universal health coverage, using both the indicator of service coverage and the indicator of financial protection, and putting into motion the close monitoring many believe is necessary for progress towards universal health coverage.^26^ Thirdly, with its broad portfolio including education, water and sanitation, and transport, the bank can work across sectors to address broader determinants of healthcare access. Finally, with these financial, logistical, and scientific resources on hand, it can leverage the money needed to promote transformative health policy.

However, at least two major risks accompany these potential benefits. Firstly, the bank has been criticised for promoting a diminished role for the state and encouraging the private sector in public health efforts in the global south, through strategies such as structural adjustment programmes and the introduction of user fees, which has increased inequality in some of the poorest countries in the world.^22^ The move towards funding private hospitals, clinics, and health insurance (such as the Health in Africa initiative) has also been criticised for not reaching those most in need.^27^

Secondly, the bank’s mandate to create new markets is often in tension with the fundamental concept at the heart of universal health coverage—that access to non-ruinous, quality healthcare is a human right. The idea of universal health coverage is firmly rooted in the right to health, set out in the International Covenant on Economic, Social, and Cultural Rights.^28^ The bank’s Articles of Agreement, however, state explicitly that it shall not be involved politically with member states and only be motivated by technical and economic considerations. Because of the bank’s role in the promotion of universal health coverage, how it manages this tension will be critical to achieving global access to equitable healthcare.

## Universal health coverage: technical or political goal?

At the start of the 21st century, the push for universal health coverage seems stronger than ever. The new WHO director general, Tedros Adhanom Ghebreyesus, asserts that the global health community is recommitted to health as a human right with universal health coverage, echoing the commitments made in 1948 and 1978.^29^ Over the past decade, WHO and the World Bank have pointed to a lack of metrics for strengthening health systems as hampering efforts. In fact, in a 2013 speech, bank president Jim Kim argued that the Alma-Ata Declaration’s Health for All goal can be achieved in the contemporary era because we have the capacity to measure success towards that goal.^30^ Yet, while global efforts and consensus towards the importance of universal health coverage are crucial, the real driver of change will come from national stakeholders such as health workers demanding the right to health and pressuring governments to find the mechanisms to deliver this goal. Such political movements have already happened all over the world—such as the launch of Senegal’s *Agence de la couverture maladie universelle* in 2013, on the heels of years of health union engagement; Thailand’s Universal Coverage Scheme, established in 2001; and Brazil’s Unified Health System, established in 1988.^31^ ^32^ ^33^

## Notes

Contributors and sources: MT collected the data, analysed it, and drafted the initial version of the paper. DS helped conceptualise and design the study and revised the draft. Competing interests: We have read and understood BMJ policy on declaration of interests and have no relevant interests to declare. This work was supported by the Wellcome Trust [106635/Z/14/Z]. A senior member of the World Bank is on our project’s advisory board.

Provenance and peer review: Commissioned; externally peer reviewed.
